# Results from the BETTER WISE trial: a pragmatic cluster two arm parallel randomized controlled trial for primary prevention and screening in primary care during the COVID-19 pandemic

**DOI:** 10.1186/s12875-023-02159-6

**Published:** 2023-09-28

**Authors:** Donna Patricia Manca, Carolina Fernandes, Aisha Lofters, Kris Aubrey-Bassler, Melissa Shea-Budgell, Denise Campbell-Scherer, Nicolette Sopcak, Christopher Meaney, Rahim Moineddin, Kerry McBrien, Paul Krueger, Tracy Wong, Eva Grunfeld

**Affiliations:** 1 Department of Family Medicine, University of Alberta, 6-10 University Terrace, Edmonton, AB T6G 2T4 Canada; 2 Covenant Health, Grey Nuns Community Hospital, 1100 Youville Drive Northwest, Edmonton, AB T6L 5X8 Canada; 3 Department of Family and Community Medicine, University of Toronto, 500 University Ave, Toronto, ON M5G 1V7 Canada; 4 Discipline of Family Medicine, Memorial University of Newfoundland, 300 Prince Phillip Drive, St. John’s, Newfoundland, A1B 3V6 Canada; 5 Charbonneau Cancer Institute and Department of Oncology, University of Calgary, 3280 Hospital Drive NW, Calgary, AB T2N 4Z6 Canada; 6 Office of Lifelong Learning & Physician Learning Program, University of Alberta, 2-590 Edmonton Clinic Health Academy, Edmonton, AB T6G 1C9 Canada; 7 Departments of Family Medicine and Community Health Sciences, University of Calgary, 3280 Hospital Drive NW, Calgary, AB T2N 4Z6 Canada; 8 Patient Advisor, Alberta Health Services, Strategic Clinical Networks, Calgary, AB Canada; 9 Ontario Institute for Cancer Research, 661 University Avenue, Suite 510, Toronto, ON M5G 0A3 Canada

**Keywords:** Cancer survivors, Chronic disease, Clinical practice guidelines, Prevention, Primary care, Screening, COVID-19, Clinical trial, Canada

## Abstract

**Background:**

Cancer and chronic diseases are a major cost to the healthcare system and multidisciplinary models with access to prevention and screening resources have demonstrated improvements in chronic disease management and prevention. Research demonstrated that a trained Prevention Practitioner (PP) in multidisciplinary team settings can improve achievement of patient level prevention and screening actions seven months after the intervention.

**Methods:**

We tested the effectiveness of the PP intervention in a pragmatic two-arm cluster randomized controlled trial. Patients aged 40–65 were randomized at the physician level to an intervention group or to a wait-list control group. The intervention consisted of a patient visit with a PP. The PP received training in prevention and screening and use of the BETTER WISE tool kit. The effectiveness of the intervention was assessed using a composite outcome of the proportion of the eligible prevention and screening actions achieved between intervention and control groups at 12-months.

**Results:**

Fifty-nine physicians were recruited in Alberta, Ontario, and Newfoundland and Labrador. Of the 1,005 patients enrolled, 733 (72.9%) completed the 12-month analysis. The COVID-19 pandemic occurred during the study time frame at which time nonessential prevention and screening services were not available and in-person visits with the PP were not allowed. Many patients and sites did not receive the intervention as planned.

The mean composite score was not significantly higher in patients receiving the PP intervention as compared to the control group. To understand the impact of COVID on the project, we also considered a subset of patients who had received the intervention and who attended the 12-month follow-up visit before COVID-19. This assessment demonstrated the effectiveness of the BETTER visits, similar to the findings in previous BETTER studies.

**Conclusions:**

We did not observe an improvement in cancer and chronic disease prevention and screening (CCDPS) outcomes at 12 months after a BETTER WISE prevention visit: due to the COVID-19 pandemic, the study was not implemented as planned. Though benefits were described in those who received the intervention before COVID-19, the sample size was too small to make conclusions. This study may be a harbinger of a substantial decrease and delay in CCDPS activities under COVID restrictions.

**Trial registration:**

ISRCTN21333761. Registered on 19/12/2016. http://www.isrctn.com/ISRCTN21333761.

## Background

Cancer and chronic diseases are a major cost to the healthcare system [1]. Many of these can be prevented or detected early to improve outcomes [1]. The majority of these can be addressed in the primary care setting [2–4]. Unfortunately, traditional medical models of care and approaches aimed to improve prevention and screening, such as the annual checkup, may not be effective [5, 6]. For example, applying the US Preventive Service Task Force recommendations was estimated to add an additional 7.4 h to a primary care physician’s day in 2003 [5, 6]. This was revisited in 2022 and the additional time required for preventive care is now 14.1 h in a day [7]. Since periodic preventive health visits and general health checks are not effective [5, 8, 9], other approaches to prevention and screening are recommended. In Canada, the model of care in primary care is evolving towards the patient’s medical home, a multidisciplinary team model [10]. This model of care is a shift away from the traditional solo practitioner or physician centric model [10]. The shift involves a change in how physicians are organized and paid and these multidisciplinary models have demonstrated improvements in chronic disease management and prevention [11, 12]. However, effective approaches to cancer and chronic disease prevention and screening in the multidisciplinary team model are needed to improve outcomes and the sustainability of our healthcare system [1, 4, 13, 14].

The BETTER trial developed an approach to primary prevention and screening for cancer and chronic disease [4]. This approach was demonstrated to be effective in improving individual patient outcomes such as optimizing diet, exercise, and blood pressure at seven months follow-up, including for patients with moderate mental illness [4, 15]. The BETTER approach involved the use of harmonized prevention and screening recommendations tailored to the patient’s personal medical history, family history, and lifestyle factors, and supported with tools and resources [16, 17] including an enhanced role within the primary care setting, the Prevention Practitioner (PP) [18, 19]. The PP was a member of the primary care multidisciplinary team who developed additional skills in cancer and chronic disease prevention and screening. The BETTER program was shown to facilitate prevention and screening in multidisciplinary team settings and has been included as a resource in the patient’s medical home model toolkit [10]. Qualitative research has also found that the effective BETTER approach was perceived to address patients’ prevention and screening, including picking up things that were missed and identifying health concerns that the physician was not aware of, such as alcoholism [19]. Numerous projects have explored and expanded on the BETTER program of research including a study of the approach in varied settings [20] and adapting and evaluating the approach into the public health setting [21].

The previous BETTER trial [4] assessed outcomes at seven months, however it is not known if outcomes are improved or sustained beyond this time frame. And, although follow-up care of cancer survivors for their prior cancer is relatively good, studies have shown that chronic disease prevention and screening for other chronic diseases is not as good for cancer survivors as it is for the general population [22]. In light of these findings, the BETTER WISE trial was designed to evaluate prevention and screening outcomes 12-months after the intervention, including outcomes in breast, colorectal and prostate cancer survivors. The project also included assessments for poverty, qualitative research on the intervention including implementation and feasibility, cancer surveillance outcomes, and sustainability outcomes of prevention and screening at 24-months.

The primary objective was to determine if patients 40–65 years of age, including cancer survivors (breast, colorectal, and/or prostate), randomized to receive the BETTER approach which included an individualized prevention visit with a PP, had improved general prevention and screening outcomes and cancer surveillance 12 months after the initial prevention visit as compared to standard care in a wait-list control group. During the trial, the corona virus disease (COVID-19) resulted in a public health state of emergency being declared in Alberta, Newfoundland & Labrador, and Ontario in March 2020 resulting in all non-essential services being closed. The aim of this paper is to present the results of the BETTER WISE trial, specifically the 12-month prevention and screening outcomes, and to describe how the trial was impacted by the COVID-19 pandemic [6]. The results for the cancer survivor population and other aspects of the project are reported separately [23–25].

## Methods

### Study design

The trial utilized a mixed methods approach using a pragmatic 2-arm cluster randomized controlled trial (RCT) on a binary composite endpoint. The trial was conducted in the primary care setting with the primary care physicians’ (PCP) practices as the unit of randomization and the individual patients as the unit of analysis. Details are published in the protocol paper [6]. The impact of the COVID-19 pandemic on the quantitative results of this study is also discussed.

Trial oversight was provided by the BETTER WISE team. Ethics approvals were obtained prior to the commencement of the trial. The trial was approved by the University of Alberta’s Health Research Ethics Board, St. Michael’s Hospital Research Ethics Board, Oak Valley Health Research Ethics Board, and Newfoundland and Labrador Health Research Ethics Board.

#### Important changes—impact of COVID on trial procedures

Patient recruitment occurred from January 2018 to August 2019. The first patient visits were held on September 2018 to August 2019. The 6-month follow-up was finalized in March 2020, and the 12-month follow-up was finalized in September 2020.

Changes to the protocol were introduced after the trial commenced alongside the COVID-19 imposed restrictions at both the practice/provider level and patient level. COVID-19 had an impact on the ability of participating clinic sites to provide ongoing committed PP resources to the study. Many PPs were required to provide other COVID-19-related care services which impacted their ability to perform the duties and activities outlined in the PP role. In Newfoundland & Labrador, we recruited three sites; however only two sites were able to stay engaged throughout the project as one site did not follow the protocol due to staff shortages. Ontario originally had four sites involved; however, one site was unable to identify a PP to do the intervention after the first PP left the clinic and another could not follow protocol due to staff redeployment to COVID-19 activities. For many sites, the 12-month visit occurred 18 months after baseline. As of March 2020, a public health state of emergency was declared in Alberta, Ontario, and Newfoundland & Labrador. During this time all non-essential services were closed, impacting patients’ ability to obtain prevention and screening services. This required a change in protocol from in-person visits with the PP to virtual PP visits to continue with the study. Patient outcomes were also impacted as patients were no longer able to obtain recommended screening (e.g., paps, mammograms, etc.) or attend services to address prevention (e.g., dietary and exercise advice, alcohol cessation, etc.).

### Participants

The setting was primary care practices in the Canadian provinces of Alberta, Ontario, and Newfoundland and Labrador. We aimed to recruit 16 primary care practices with 4 PCPs per practice for a total of 64 PCPs (32 in Alberta, 16 in Ontario, 16 in Newfoundland & Labrador). A two-arm cluster RCT design was used and participating PCPs were randomized to have their patients in the intervention group or wait-list control group as detailed in the BETTER WISE protocol [6]. The PCP was defined as the “cluster” to minimize the risk of contamination between patients, such that all patients in that physician cluster received the intervention or wait-list control [26].

We targeted patients in the 40 to 65 age group since most chronic disease prevention and screening activities are applicable to this group [4, 6]. All eligible breast, colorectal, and/or prostate cancer survivors were also invited; the remainder of the sample was designated as general population patients. We planned to recruit 20 patients per PCP (five cancer survivors and 15 general population patients), with priority given to cancer survivors in order maximize power for the cancer survivor specific outcome.

#### Inclusion criteria

Eligible patients included those aged 40–65 for whom we had medical record access for the previous three years as described in our protocol [6]. We required access to the previous three years of medical records to identify and review prevention and screening targets. Within this sample, we also targeted and stratified as: 1) cancer survivors (breast, colorectal, prostate) on active surveillance for recurrence (i.e., are not palliative or undergoing active treatment) and 2) general population patients (i.e., who do not have a history of breast, colorectal, or prostate cancer).

#### Exclusion criteria

Patients were excluded if they: 1) received care from another care group (e.g., nursing home patients), 2) were receiving palliative or end-of-life-care, 3) were receiving active treatment for cancer, or 4) were unable to give written informed consent. Patients undergoing prophylactic or hormone treatments (e.g., aromatase inhibitors) were not excluded.

#### Identifying the general population patient sample

The PPs and/or primary care clinic staff reviewed their patient records to identify patients who were not breast, colorectal, and/or prostate cancer survivors and generated a list of eligible patients for each participating PCP. The study biostatistician generated a random number sequence and patients were invited in that order to participate and be assigned a unique study identification (ID) number.

#### Recruitment

Standardized invitation letters were signed by the PP and PCP and mailed to patients. As stated in the letter, the participating primary care site called the patient five business days after mailing the invitation to determine if they were interested in participating. If the patient met the inclusion criteria, a verbal consent process was completed.

### Interventions

Each practice setting identified a clinician (e.g., clerk, licensed practical nurse, registered nurse, nurse practitioner, dietitian) to take on the PP role and obtain skills in cancer and chronic disease prevention and screening (CCDPS) and use of the BETTER WISE tools [6]. The BETTER WISE tools were derived from a high-level evidence review and synthesis. The toolkit included: a health survey, a care path for prevention and screening, a care path for cancer surveillance, a prevention prescription, S.M.A.R.T. (specific, measurable, attainable, realistic, time-bound) health goals, and a Bubble diagram to illustrate the patients’ prevention and screening status. The intervention consisted of a one-hour prevention visit with the PP.

*Intervention group:* Patients randomized to the intervention were scheduled for a 60-min prevention visit with their PP. Patients were asked to complete the BETTER WISE health survey prior to the visit and at six-month intervals up to 24 months after the initial visit. Intervention patients had the opportunity to review their written consent form online prior to beginning the health survey. Written consent was obtained by the PP or another clinic staff member before the patient’s first prevention visit.

Before the visit, the PP reviewed the patient’s health survey and the medical chart to determine which prevention and screening actions the patient was eligible to receive and to prepare to discuss the patient’s risk for chronic diseases such as cancer, diabetes, and heart disease. Using a shared decision-making process, the PP and the patient developed a personalized prevention prescription. The prevention prescription was written on the standardized BETTER WISE form and included plans for screening and referrals to programs (e.g., smoking cessation).

*Wait-list control group:* Wait-list control patients were mailed: 1) the website link to the BETTER WISE health survey, and 2) the study consent form to be completed and returned to the study team using a pre-addressed, stamped envelope. Wait-list control patients were asked to complete the health survey immediately after agreeing to participate in the study and before their first visit with the PP, which was scheduled upon completion of the intervention period of the study approximately 12 months later. Additional screening and prevention data was obtained for wait-list control patients by the PP or primary care clinic staff from the patient’s medical chart at baseline and 12-months.

*All patients:* If access to a computer or the internet was identified as a barrier to participation, primary care sites used alternative methods (e.g., a paper survey or iPad) to enable patients to participate in the study.

### Outcomes

A composite index [27] was used to determine if general prevention and screening outcomes improved for patients who received an individualized visit with a PP, when compared to wait-list controls at 12 months follow-up. The composite index was calculated as the total number of CCDPS actions completed at 12-months divided by the total number of CCDPS actions the patient was eligible to receive/complete at baseline, multiplied by 100. The composite index was treated as a continuous outcome, expressed as a percentage that ranged from 0 to 100 and calculated at the patient level. We revised and updated the composite index developed for CCDPS from the BETTER trial [4, 28] to reflect the clinical evidence and CCDPS messages and actions recommended in each participating province to be consistent with jurisdiction-specific messaging at the time of the study. The primary outcome and performance indicator was a composite of 24 evidence-based chronic disease prevention and screening actions related to diabetes, cardiovascular disease, cancer screening, and lifestyle factors associated with those chronic diseases. The initial planned analysis was to adhere to the intention-to-treat principle [6]. The unit of analysis was the individual patient. A secondary exploratory analysis of the impact of COVID involved a per protocol analysis.

### Sample size

We conservatively estimated the intra-class correlation coefficient (ICC) to be 0.30 based on our previous research [4, 6, 20]. From the BETTER trial, we anticipate the percentage of achieved CCDPS actions in the wait-list control arm would be approximately 20% [4]. To detect a 20% intervention effect (i.e., the proportion of achieved CCDPS actions for patients randomized to the BETTER WISE intervention is 40%, compared to 20% for wait-list control) with 80% power, we aimed to recruit four patients per PCP to achieve our sample size requirements per area of interest (cancer survivors and general population patients) at 5% alpha. The 20% intervention effect size is conservative given information gathered from the BETTER trial [4]. As such, this study required four cancer survivors and four general population patients for each participating PCP to have sufficient power to conduct sub-group analyses (i.e., measuring the effectiveness of the PP intervention in cancer survivors and the general population patients).

To account for additional planned sub-analyses and possible study withdrawal and/or loss to follow-up, we further inflated our sample size for a total of 20 patients per PCP (five cancer survivors and 15 general population patients) for a total sample size of 1,280 patients (320 cancer survivors and 960 general population patients). We planned to recruit a total of 30 patients per PCP to ensure our target sample size of 20 patients per PCP was met (five cancer survivors and 15 general population patients).

### Randomization

The biostatistician randomized the physicians within a practice to the intervention or wait-list control. Randomizing at the physician level minimized the risk of contamination in that all patients in that physician cluster received the intervention or the wait-list control. However, risk of contamination did remain as some of the patients within the same practice would receive the intervention while others the wait-list control. Despite that, we perceived this risk to be low based on our previous BETTER Trial experience.

### Statistical methods

The study used a cluster randomized controlled trial design. Both simple means, as well as generalized estimating equation models, with compound symmetric working correlation structure, were used to estimate the difference in accomplishment of our composite outcome between groups randomized to the PP intervention arm versus the wait-list control arm. Participants who were missing 12-months follow-up information used for estimation of the composite outcome measure were excluded from our primary analysis. For primary study objectives, we analyzed groups (with non-missing baseline/follow-up data) as randomized using a modified ITT principle; whereas for secondary objectives related to COVID-19 pandemic impacts on trial implementation we also present per protocol estimates of the PP intervention effect (the per-protocol sub-analyses acknowledge that some sites had trouble implementing the PP intervention during the pandemic). To evaluate demographic and clinical characteristics associated with differences in composite outcome score, estimates of the adjusted impact of intervention/PP after controlling or accounting for other variables in the linear GEE model (with compound symmetric working correlation structure) were used.

## Results

**Fig. 1:**
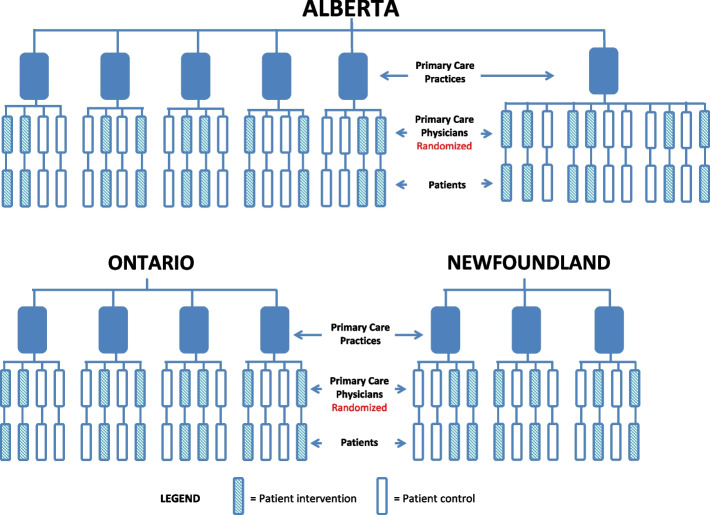
BETTER WISE design schema

We were ultimately able to recruit 13 practices. We recruited 11 PCPs in one large practice and four PCPs in the remaining 12 practices for a total of 59 PCPs, of which 30 PCPs were allocated to the intervention group and 29 PCPs to the wait-list control group (see design schemas Fig. 1). Each practice identified an individual to assume the role of the PP.

### Patients

**Fig. 2:**
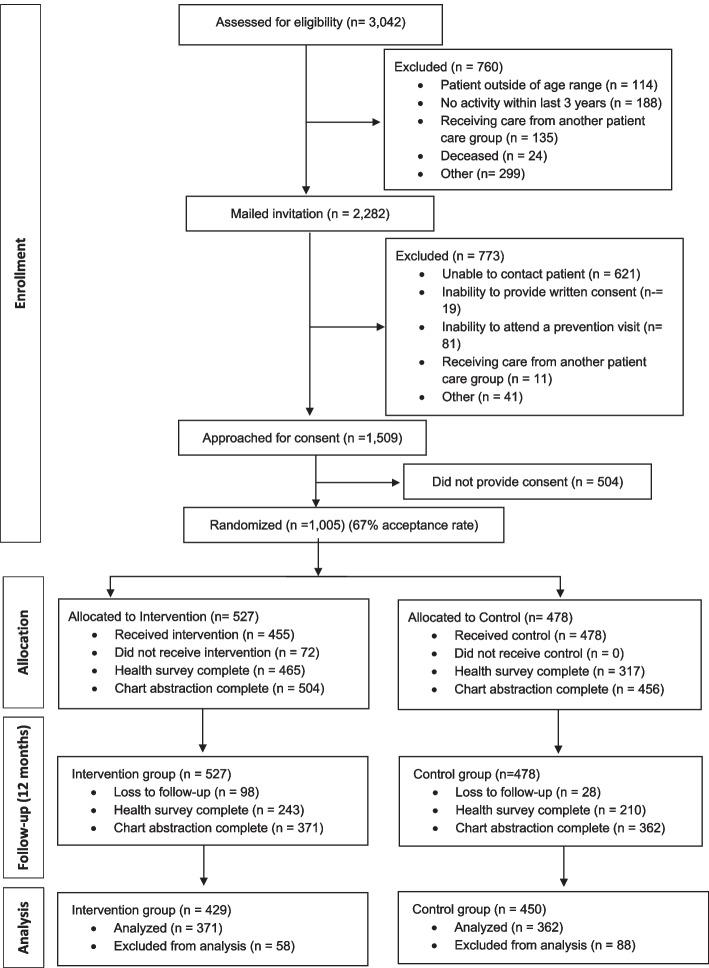
BETTER WISE consort flow diagram

One-thousand-five patients provided consent and were enrolled in the trial out of 1,509 patients approached, representing an acceptance rate of 66.6%. Of the 1,005 patients enrolled, 733 (72.9%) were retained for the 12-month analysis. The return rate at the 12-month follow-up visit was 371/527 (70.4%) for the intervention group and 362/478 (75.7%) for the control group. Details are outlined in the CONSORT diagram (Fig. 2). Of the patients enrolled, 115 were cancer survivors. Baseline and follow-up data was available for 80 of these patients. Quantitative findings for this group are reported elsewhere [23].

Baseline demographic characteristics are illustrated in (Table 1). The control and intervention group are similar, however there was a higher amount of missing data in the control group.

**Table 1:** Baseline characteristics of patients by randomization group (*N* = 733). Data are N (%) unless otherwise stated

	**Control** **(*N* = 362)**	**Intervention** **(*N* = 371)**	**SMD**
Age – yr ± SD	54.0 ± 7.0	54.5 ± 7.0	0.066
**Sex**			
Female	218 (60.2)	242 (65.2)	
Male	144 (39.8)	129 (34.8)	
Missing	0	0	0.104
**Ethnic background**			
Caucasian	213 (82.9)	278 (84.5)	
Non-Caucasian	44 (17.1)	51 (15.5)	0.044
Missing	105	42	
**Education**			
≥ 1-year post-secondary education	194 (72.7)	269 (78.2)	
< 1-year post-secondary education	73 (27.3)	75 (21.8)	
Missing	95	27	0.129
**Employment**			
Full-time or part-time	193 (73.1)	249 (72.8)	
Other	71 (26.9)	93 (27.2)	
Missing	98	29	0.007
**Marital status**			
Married or common-law	219 (82.6)	277 (80.5)	
Other	46 (17.4)	67 (19.5)	
Missing	97	27	0.055
**Total household income**			
< $60,000 CAD	47 (21.2)	86 (29.2)	
$60,000 – 99,999 CAD	67 (30.2)	63 (21.4)	
$100,000 – 149,999 CAD	54 (24.3)	70 (23.7)	
> $150,000 CAD	54 (24.3)	76 (25.7)	
Missing	140	76	0.239
**Smoking status**			
Current smoker	31 (11.8)	51 (14.8)	
Not Current smoker	232 (88.2)	293 (85.2)	
Missing	99	27	0.090
**Alcohol consumption**			
More than recommended	16 (7.9)	27 (10.2)	
Within recommended amounts	187 (92.1)	237 (89.8)	
Missing	159	107	0.082
**Physical activity**			
≥ 150 min per week	65 (25.6)	70 (21.0)	
< 150 min per week	189 (74.4)	264 (79.0)	
Missing	108	37	0.110
**Body mass index (BMI)—mean ± SD**	29.5 ± 6.8	28.8 ± 5.8	0.099
18.5 – 24.9	81 (24.1)	83 (23.1)	
25.0 – 29.9	114 (34.0)	128 (35.6)	
30 – 34.9	93 (27.7)	88 (24.4)	
35 – 39.9	24 (7.1)	35 (9.7)	
≥ 40	24 (7.1)	26 (7.2)	
Missing	26	11	0.115
**PHQ-2 score—mean ± SD**	0.8 ± 1.3	0.9 ± 1.2	0.041
Positive screen	26 (9.7)	28 (8.1)	
Negative screen	241 (90.3)	318 (91.9)	
Missing	95	25	0.058
**GAD-2 score – mean ± SD**	1.1 ± 1.5	1.1 ± 1.4	0.019
Positive screen	34 (12.8)	39 (11.4)	
Negative screen	232 (87.2)	303 (88.6)	
Missing	96	29	0.042
**Trouble making ends meet**			
Yes	55 (20.8)	69 (20.8)	
No	210 (79.2)	262 (79.2)	
Missing	97	40	0.002
EQ-5D-5L score – mean ± SD	0.8 ± 0.2	0.8 ± 0.1	0.034
Follow-up time—days ± SD	447.0 ± 96.2	402.4 ± 78.3	0.509

*SD* Standard deviation, *SMD* Standardized mean difference, Body mass index (BMI) is the weight in kilograms divided by the square of the height in meters. PHQ-2: Patient Health Questionnaire 2. The PHQ-2 includes the first two items of the PHQ-9 and is a brief screening tool for depression. Scores range from 0 to 6. A score of 3 or more points is a positive screen for possible depression [29]. GAD-2: Generalized Anxiety Disorder 2-item. The GAD-2 is a brief and easy to perform initial screening tool for generalized anxiety disorder. Scores range from 0 to 6. A score of 3 or more points is a positive screen for possible generalized anxiety disorder [30]. EQ-5D-5L: A preference-based health related quality of life measure. The EQ-5D-5L consists of the EQ-5D descriptive system and the EQ visual analogue scale. The descriptive system includes five dimensions (1—mobility, 2—self-care, 3—usual activities, 4—pain and discomfort, and 5—anxiety and depression), each of which has five severity levels. Patients’ responses can be combined to generate a 5-digit number that describes their health state [31]

The baseline eligibility for each of the 24 prevention and screening actions are illustrated in Table 2.

**Table 2:** Baseline eligibility of patients for prevention and screening actions by randomization group

	**Control (*N* = 362)**		**Intervention (*N* = 371)**		**SMD**
**Prevention and screening actions (*N* = , eligible)_**	**N**	**%**	**N**	**%**	
1. Fasting blood sugar or hemoglobin A1c screening	201	55.7	176	47.4	0.165
2. Fasting blood sugar or hemoglobin A1c monitoring	20	5.5	10	2.7	0.144
3. Blood pressure screening	133	36.8	87	23.5	0.295
4. Blood pressure monitoring	76	21.1	62	16.7	0.111
5. Breast cancer screening (women only; *N* = 449)	80	36.9	77	32.0	0.104
6. Colorectal cancer screening	98	27.1	98	26.4	0.015
7. Cervical cancer screening (women only; *N* = 449)	97	44.5	58	24.0	0.443
8. Cardiovascular risk assessment	116	32.1	74	20.0	0.279
9. ACE or ARB optimization referral	5	1.9	11	3.2	0.083
10. BMI Screening	221	61.0	184	49.6	0.232
11. Waist circumference measurement	113	33.6	122	33.9	0.005
12. Cholesterol treatment	36	10.0	41	11.1	0.035
13. Weight control referral	141	42.0	149	41.4	0.012
14. Smoking cessation referral	31	11.8	51	14.8	0.09
15. Alcohol cessation referral	49	23.6	68	25.5	0.044
16. Physical activity referral	189	74.4	264	79.0	0.110
17. Nutrition/Diet referral	240	89.6	307	88.5	0.035
18. Hypertension control	105	29.1	110	29.6	0.012
19. Depression score improvement	26	9.7	28	8.1	0.058
20. At-risk alcohol improvement	101	48.6	144	53.7	0.104
21. Low physical activity improvement	189	74.4	264	79.0	0.110
22. Overweight improvement	255	75.9	278	77.2	0.031
23. Smoking cessation	31	11.8	51	14.8	0.090
24. Healthy diet score improvement	240	89.6	307	88.5	0.035

*SMD* Standardized mean difference, *ACE* Angiotensin converting enzyme inhibitor. Used to prevent, treat, or improve symptoms in conditions such as high blood pressure (hypertension), heart failure, and diabetes [32]. ARB: Angiotensin-receptor blockers. Used to treat high blood pressure and prevent, treat, or improve symptoms in people with chronic conditions such as heart failure [32]. *BMI* Body mass index is the weight in kilograms divided by the square of the height in meters

The mean number of CCDPS actions for which patients were eligible at baseline was 7.7 in the control group and 8.1 in the intervention group as illustrated in Table 3. The mean composite score was 28.6 in the control group and 27.6 in the intervention group (*p* = 0.845) (Table 3).

**Table 3:** Prevention and screening actions by randomization group. Data are Mean ± SD

	**Control** **(*N* = 362)**	**Intervention** **(*N* = 371)**	*P* value*
**All patients; N**			
Eligible actions	7.7 ± 3.0	8.1 ± 2.8	0.207
Actions met	2.2 ± 1.8	2.3 ± 2.1	0.650
Composite score	28.6 ± 22.5	27.6 ± 24.0	0.845

Composite score: defined as the ratio of the number of cancer and chronic disease prevention and screening actions met according to pre-defined targets to the number of actions for which the patient was eligible

^*^P values are based on two-sided Wald tests using robust covariance estimate

The PP intervention effect was small and not statistically significantly different from the null after adjustment for potential confounding factors, as shown in Table 4 (Δ = 0.03; 95% CI = -0.01 to 0.08; *p* = 0.163). In the province of Alberta, female sex, age over 60, other non-Caucasian ethnic backgrounds, and no post-secondary education were associated with a more positive composite outcome as illustrated in Table 4.

**Table 4:** Demographic and clinical characteristics associated with differences in composite outcome score (*N* = 578)

	**Δ Outcome (95% CI)**	*P* value*
**Intercept**	0.27 (0.22, 0.33)	< 0.001
**Randomization arm:** control	reference	-
Intervention	0.03 (-0.01, 0.08)	0.163
**Province:** Alberta	reference	-
Newfoundland & Labrador	-0.22 (-0.28, -0.16)	< 0.001
Ontario	-0.20 (-0.26, -0.14)	< 0.001
**Sex:** Male	reference	-
Female	0.04 (0.01, 0.08)	0.018
**Age:** 40 – 44 years old	reference	-
45 – 49 years old	0.04 (-0.02, 0.09)	0.201
50 – 54 years old	0.07 (0.01, 0.12)	0.023
55 – 60 years old	0.05 (-0.01, 0.10)	0.116
> 60 years old	0.08 (0.02, 0.14)	0.013
**Ethnic background:** Caucasian	reference	-
Other ethnic background	-0.07 (-0.12, -0.02)	0.008
**Education:** ≥ 1-year post-secondary	reference	-
No post-secondary education	0.05 (0.01, 0.09)	0.027
**GAD-2:** Negative screen	reference	-
Positive screen	0.03 (-0.01, 0.08)	0.156

A fitted generalized estimating equation model with compound symmetric working correlation structure was used to estimate the parameters of the multivariate model. Data are expected difference in composite outcome relative to the reference condition for the variable. *CI* Confidence interval, *GAD-2* Generalized Anxiety Disorder 2-item. The GAD-2 is a brief and easy to perform initial screening tool for generalized anxiety disorder. Scores range from 0 to 6. A score of 3 or more points is a positive screen for possible generalized anxiety disorder [30]

^*^P values are based on two-sided Wald tests using robust covariance estimate

### COVID-19

The COVID-19 pandemic occurred during the study time frame. Nonessential prevention and screening services such as screening tests and in-person visits for prevention services (e.g., dietitians, exercise, etc.) were no longer available. In-person visits with the PP were not allowed during this time, so the study adapted, and prevention visits were provided virtually or over the telephone. Due to COVID many patients and sites did not receive the intervention as planned. To explore and better understand the impact of COVID on the trial we considered a subset of patients who had received the intervention and who attended the 12-month follow-up visit pre-COVID-19 (i.e., visits that took place before February 2020). We considered using intention-to-treat analysis; however, it does not consider those patients and sites that were not able to implement the intervention as planned due to COVID-19. We therefore performed a per-protocol analysis to explore the outcomes in those patients who received the intervention as planned. This information is illustrated in Table 5.

**Table 5:** The impact of the COVID-19 pandemic on the composite outcome score by randomization group

Control		Intervention		***P***-value*
**Patients (N)**	**Composite Score (Mean, 95% CI)**	**Patients** **(N)**	**Composite Score (Mean, 95% CI)**	
67	21.3 (13.2, 29.3)	69	42.1 (30.8, 53.4)	0.003

Composite score: defined as the ratio of the number of cancer and chronic disease prevention and screening actions met according to pre-defined targets to the number of actions for which the patient was eligible. Data are difference in composite outcome relative to the reference condition for the variable. *CI* confidence interval

^*^P values are based on two-sided Wald tests using robust covariance

Though the overall results demonstrate a null trial, we found a significant difference in a subset of 136 participants before the impact of COVID-19. There was a significant improvement in the composite score of the intervention group (42%) as compared to the control group (21%) *p* = 0.003.

## Discussion

Effective approaches for cancer and chronic disease prevention are required for improved health outcomes and sustainability of the healthcare system [1, 4, 13, 14]. The BETTER approach has been demonstrated to effectively improve patients’ prevention and screening outcomes in primary care team settings [4, 20]. The approach consists of enhancing the role of a member of the primary care setting to become a PP. The PP develops skills and dedicates some time to prevention and screening visits with patients. The PP is provided with and educated on the use of the BETTER toolkit.

The BETTER WISE trial was an ambitious mixed methods project that aimed to evaluate prevention and screening outcomes 12-months after the intervention including sustainability at 24-months in both the general population and cancer survivors. The project also involved knowledge synthesis of high-level guidelines to develop the resources and the BETTER WISE toolkit used by the PP [23] as well as a qualitative evaluation that employed a constant comparative method to describe what a prevention visit is [19] and understand the facilitators and barriers to implementing the BETTER WISE approach [24].

The BETTER approach has demonstrated to be an effective approach to CCDPS in multidisciplinary team settings [4], and in varied settings including rural sites [20] at approximately half a year after the intervention. In contrast, The BETTER WISE trial did not demonstrate effectiveness one year after the intervention. The PP intervention is dependent on an integrated model of care; that is, a multidisciplinary team model with access to primary prevention and screening resources. When access to nonessential resources was shut down due to the COVID-19 pandemic in Canadian jurisdictions, the BETTER WISE intervention was no longer possible. We were able to adapt so that PPs were able to hold virtual/telephone prevention visits with patients; however, this did not replace the team-based care that also suffered due to redeployment of healthcare resources to COVID-19 priorities. Patients were no longer able to access resources such as laboratories, mammograms, dietitians, exercise facilities, and more. Patients in the intervention group did not improve their prevention and screening as evaluated by a composite index that included assessments of lifestyle factors such as diet, exercise, smoking, and alcohol consumption. However, we describe an improvement in prevention and screening outcomes in a small number of participants before the impact of COVID-19, though the sample size is too small to make conclusions. The COVID-19 pandemic has impacted the health of Canadians and this has been documented in other studies that demonstrate a delay in cancer screening [33] and a deterioration of healthy behaviours [34].

The limitations of this study include the following: 1) inability to implement the study as planned due to the COVID-19 pandemic, resulting in lack of access to prevention and screening resources and change from in-person to virtual or telephone visits; 2) changes in the ability of the sites to provide ongoing committed PP resources due to a need to re-allocate services and resources to address the COVID-19 pandemic; and 3) patients dropping-out and missing data. Also, because participating PCPs were nested in a site, and some patients within a single clinical site received the intervention while others did not, there remained a risk of contamination. However, we perceived this risk to be low based on our previous experience with the BETTER trial [4, 6]. Further, if contamination did occur, it would bias estimated effects towards the null hypothesis [6].

Suggestions for future studies include assessment of virtual preventive visits. In our study, virtual visits did not mitigate against the downstream system impacts of COVID; however they may be effective in a multidisciplinary approach where COVID-related system closures or redeployments are not in place. In support of this, our pre-COVID per-protocol analysis demonstrated the effectiveness of the BETTER visits, similar to the findings in previous BETTER studies [4, 20].

## Conclusions

We did not observe an improvement in CCDPS outcomes at 12 months after a BETTER WISE prevention visit and the study was not implemented as planned due to the COVID-19 pandemic. Benefits may be observed in settings not impacted by imposed restrictions to preventive care and resources. This study may be a harbinger of potential increase in the incidence of chronic disease and cancer due to a substantial decrease and delay in CCDPS activities under COVID restrictions.

## Data Availability

The datasets generated during and/or analyzed during the BETTER WISE study will not be made publicly available due to planned analyses and publications but are available from the corresponding author on reasonable request. We will not provide full transcriptions of the qualitative data as they may contain quotes and identifiable information that could compromise the identity of participants.

## Abbreviations

BETTER: Building on Existing Tools to Improve Chronic Disease Prevention and Screening in Primary Care
BETTER trial: Building on Existing Tools to Improve Chronic Disease Prevention and Screening in Primary Care; a pragmatic randomized controlled trial in Alberta and Ontario
BETTER 2: Building on Existing Tools to Improve Chronic Disease Prevention and Screening in Primary Care; an implementation study in Newfoundland & Labrador and the Northwest Territories
BETTER WISE: Building on Existing Tools to Improve Cancer and Chronic Disease Prevention and Screening in Primary Care for Wellness of Cancer Survivors and Patients
COVID or COVID-19: Corona virus disease
CCDPS: Cancer and chronic disease prevention and screening
EPICORE: Epidemiology Coordinating and Research Centre
ICC: Intraclass correlation coefficient
ID: Identification
ITT: Intention to treat
PCP: Primary care provider
PP: Prevention Practitioner
RCT: Randomized controlled trial
REDCap: Research electronic data capture
SMART: Specific, measurable, attainable, realistic, time-bound
